# Zika Virus Fatally Infects Wild Type Neonatal Mice and Replicates in Central Nervous System

**DOI:** 10.3390/v10010049

**Published:** 2018-01-22

**Authors:** Shuxuan Li, Najealicka Armstrong, Huan Zhao, Wangheng Hou, Jian Liu, Chunye Chen, Junkai Wan, Wei Wang, Chunlian Zhong, Che Liu, Hua Zhu, Ningshao Xia, Tong Cheng, Qiyi Tang

**Affiliations:** 1 State Key Laboratory of Molecular Vaccinology and Molecular Diagnostics, National Institute of Diagnostics and Vaccine Development in Infectious Diseases, School of Life Sciences, Xiamen University, Xiamen 361102, China; lishuxuan37@163.com (S.L.); zh373183889@163.com (H.Z.); houwangheng@xmu.edu.cn (W.H.); m13113141373@163.com (C.C.); wjk927@163.com (J.W.); lukewang@xmu.edu.cn (W.W.); liuche@xmu.edu.cn (C.L.); nsxia@xmu.edu.cn (N.X.); 2 Department of Microbiology, Howard University College of Medicine, Washington, DC 20059, USA; narmstrong@Howard.edu; 3 Department of Microbiology and Molecular Genetics, New Jersey Medical School, Rutgers University, 225 Warren Street, Newark, NJ 070101, USA; jl2147@njms.rutgers.edu (J.L.); zhuhu@njms.rutgers.edu (H.Z.); 4 Department of Basic Medical Sciences, Medical College of Xiamen University, Xiamen 361102, China; zhongchunlian0117@126.com

**Keywords:** Zika virus (ZIKV), neonatal mouse, pathogenesis, microcephaly, *Flavivirus*

## Abstract

Zika virus (ZIKV) has been defined as a teratogenic pathogen behind the increased number of cases of microcephaly in French Polynesia, Brazil, Puerto Rico, and other South American countries. Experimental studies using animal models have achieved tremendous insight into understanding the viral pathogenesis, transmission, teratogenic mechanisms, and virus–host interactions. However, the animals used in published investigations are mostly interferon (IFN)-compromised, either genetically or via antibody treatment. Herein, we studied ZIKV infection in IFN-competent mice using African (MR766) and Asian strains (PRVABC59 and SZ-WIV01). After testing four different species of mice, we found that BALB/c neonatal mice were resistant to ZIKV infection, that Kunming, ICR and C57BL/6 neonatal mice were fatally susceptible to ZIKV infection, and that the fatality of C57BL/6 neonates from 1 to 3 days old were in a viral dose-dependent manner. The size and weight of the brain were significantly reduced, and the ZIKV-infected mice showed neuronal symptoms such as hind-limb paralysis, tremor, and poor balance during walking. Pathologic and immunofluorescent experiments revealed that ZIKV infected different areas of the central nervous system (CNS) including gray matter, hippocampus, cerebral cortex, and spinal cord, but not olfactory bulb. Interestingly, ZIKV replicated in multiple organs and resulted in pathogenesis in liver and testis, implying that ZIKV infection may engender a high health risk in neonates by postnatal infection. In summary, we investigated ZIKV pathogenesis using an animal model that is not IFN-compromised.

## 1. Introduction

Zika virus (ZIKV), together with West Nile virus, yellow fever virus, Japanese encephalitis virus, Dengue fever virus, and many other viruses, is a member of the genus *Flavivirus* of the family *Flaviviridae* [1,2,3]. A growing number of strains of ZIKV have been isolated from more than 60 countries [4,5]. In earlier studies, it was determined that ZIKV caused only a mild arthropod-borne disease in humans, known as Zika fever; therefore, ZIKV-related research had been neglected until the recent epidemic outbreaks and association with newborn microcephaly. ZIKV was first isolated from a monkey, and it is known that ZIKV can be transmitted to humans via a mosquito bite or occasionally by sexual contact [4,6,7,8,9]. Many years later in Nigeria, ZIKV was isolated from humans [10]. Since 2007, ZIKV has caused epidemic outbreaks with different scales in Micronesia, French Polynesia, Cook Island, and Easter Island, and has become an emerging arbovirus [11]. More recently, a pandemic of ZIKV infection occurred in South America, especially in Brazil, which prompted the World Health Organization (WHO) to declare that ZIKV-caused infection, symptoms, and complications are a public health emergency on 1 February 2016 [12]. ZIKV infection has been related to the increasing number of cases of microcephaly and Guillain–Barré syndrome (GBS) in the areas of the epidemics [4,13,14]. The recent reports that ZIKV infection probably associated with microcephaly in neonates and GBS in adults have spurred researchers to seriously reevaluate the medical significance of this agent as a pathogen [15,16,17,18].

Detailed information about ZIKV that can be acquired by deep investigation at the molecular, epidemiological, and clinical levels will be critical for illustrating its role as a pathogen of serious diseases in humans. Currently, the molecular etiology of ZIKV infection and its effects have begun to be elucidated. First, in vitro experiments showed that ZIKV infection impaired the proliferation program of neural stem cells [19,20,21,22]. Second, in vivo studies using mouse models revealed that ZIKV can pass through the placental barrier to infect fetus, and the infected fetus may die or develop microcephaly or other malformations of the brain [23,24,25,26,27]. Recent studies using mouse models demonstrated that ZIKV infection directly inhibited neuron stem cell proliferation, which supports the hypothesis that ZIKV is causatively related to microcephaly, and is consistent with the observations in humans [23,24,25,26,28,29]. In addition, epidemiological data also statistically linked ZIKV infection and its outbreaks to the increased cases of microcephaly and GBS in the area of ZIKV epidemics [13,16,30,31]. Interestingly, epidemiological and phylogenetic studies have found that the recent cases of microcephaly and GBS linked to ZIKV are mostly caused by Asian strains [32,33,34,35].

Animal models are always important for investigating the pathogenesis of the viral infection. The wild type (WT) adult mouse is resistant to ZIKV infection, although it is important for ZIKV study [26,36,37]. The mice used as models for ZIKV studies so far have deficiencies in the interferon (IFN) or IFN receptor which are caused either genetically or via antibody treatment [38]. The A129 strain mouse, lacking receptors for type I IFN (IFN alpha and beta), is not susceptible to stimulation of IFN-alpha and -beta, and is vulnerable to ZIKV infection [28]. The same results were achieved using the AG129 mouse model that lacks the IFN-alpha, -beta, and -gamma receptors [25,28]. Another strain of mouse called triple knockout (TKO), with knockout of three genes [*irf3(−/−), irf5(−/−)* and *irf7(−/−)*], produces little IFN-alpha and -beta, and is also fatally susceptible to ZIKV infection [25]. The importance of IFN-alpha/beta signaling in preventing ZIKV infection has also been confirmed using anti-IFN-alpha receptor antibody. The blockade of the type I IFN response using the anti-IFNaR1 antibody in WT pregnant mice enabled ZIKV to cross the placenta to infect the fetus [39]. Besides IFN-alpha, -beta, and -gamma receptors, it has been recently demonstrated that fetus lacking IFN-λ receptor had increased ZIKV replication in the placenta and fetus [37]. In addition, it was demonstrated that ZIKV evades IFN-mediated host anti-viral defense by targeting signal transducer and activator of transcription 2 (STAT2) [40,41], consistently, the STAT2-deficient mouse is vulnerable to ZIKV infection [42]. Thus far, the WT mice that were used for ZIKV were neonatal ones [43,44]. One-day-old WT C57BL/6 mice were shown to be infected with ZIKV and developed symptoms [44]. Here, we systemically investigated ZIKV infection in neonatal mice and present the outcomes in pathogenesis, virus-host interaction, and development of neuronal damages.

## 2. Materials and Methods

### 2.1. Mice

BALB/c, C57BL/6, ICR and Kunming (KM) mice used in this study were supplied by the Slac Laboratory Animal Co., Ltd., Shanghai, China and housed under specific-pathogen-free conditions.

### 2.2. Ethics Statement

All animal experiments were carried out in strict compliance with the Animal Welfare Act, public health service Policy and the standards of the American Association for the Accreditation of Laboratory Animal Care and other national statutes and regulations relating to animals. The animal protocol was approved by the Institutional Animal Care and Use Committee (IACUC) and Laboratory Animal Management Ethics Committee at Xiamen University (Protocol Number: XMULAC20160049) (Approved on 6 January2017).

### 2.3. Cells and Viruses

African green monkey kidney (Vero) cells (American Type Culture Collection, ATCC) were cultured in minimal essential medium (MEM, GIBCO, Gaithersburg, MD, USA) with 10% fetal bovine serum (FBS, GIBCO), 2 mM l-glutamine, 100 U/mL of penicillin and 100 μg/mL of streptomycin, and maintained at 37 °C with 5% CO_2_.

Two Asian lineage ZIKV strains, PRVABC59 (GenBank: KU501215) and SZ-WIV01 (GenBank: KU963796), and one African lineage ZIKV strain, MR766 (GenBank: LC002520) were used in this study. PRVABC59, originally isolated from the serum sample of a ZIKV-infected patient returning from Puerto Rico in 2015 [33], was purchased from ATCC. SZ-WIV01, originally isolated from the serum sample of a 38-year-old Chinese male patient who had travelled to Fiji and Samoa in 2016 [45], was kindly provided by the Academy of Military Medical Sciences of China. MR766, isolated from a sentinel monkey in Uganda in 1947 [46], was purchased from ATCC. All the three virus strains were passaged for less than five times in Vero cells in our lab. The 50% tissue culture infectious dose (TCID_50_) of the virus stocks were determined in Vero cells using the Reed–Muench method [47]. Viral RNA genomes were sequenced and found to be identical to the original ones as shown in https://www.viprbrc.org.

### 2.4. Viral Inoculation and Clinical Evaluation of Mice by Health Score

To compare the susceptibility of different species of mice to ZIKV, 1-day-old BALB/c, C57BL/6, KM, and ICR mice (*n* = 8–10 per group) mice were infected intraperitoneally (i.p.) with 10^6^ TCID_50_/mouse of PRVABC59. To compare the pathogenic effects of different ZIKVs on mice, three ZIKV strains (PRVABC59, SZ-WIV01 and MR766) were used to infect 1-day-old C57BL/6 mice (*n* = 8–10 per group) i.p. or subcutaneously (s.c.) with 10^6^ TCID_50_/mouse or intracerebrally (i.c.) with 10^5^ TCID_50_/mouse. To determine the 50% lethal dose (LD_50_) of ZIKV, 1-day-old C57BL/6 mice (*n* = 8–10 per group) were i.p. infected with a dose of 10-fold serial dilutions of PRVABC59 (from 10^0^ to 10^6^ TCID_50_/mouse), or with phosphate-buffered saline (PBS) or UV-inactivated PRVABC59 as control groups. The LD_50_ was calculated as described by Reed and Muench [46]. To define the maximum age of the neonates that could still be productively infected by ZIKV, 1-, 3-, 5-, 7-, 14- and 21-day-old C57BL/6 mice (*n* = 8–10 per group) were inoculated i.p. with PRVABC59 (10^6^ TCID_50_/mouse), or were administered with PBS as control.

Health score: Mice were observed daily for body weight and clinical signs for 25 days after infection or until their deaths. The grade of clinical signs was scored as follows: 0, healthy; 1, lethargy and inactivity; 2, wasting; 3, limb weakness; 4, hind-limb or fore-limb paralysis and tremors; and 5, moribund and death.

### 2.5. Samples Isolation from ZIKV-Infected Mice

After infecting 1-day-old C57BL/6 mice i.p. with PRVABC59 in a dose of 10^6^ TCID_50_/mouse (as the experimental mice) or PBS (as the control mice), 25 experimental mice and three control mice were subjected to virus load assays. Samples were collected at 2, 4, 6, 8, 10 and 12 days post-infection (dpi) from experimental mice and collected at 0 dpi from control mice (*n* = 3 per time point). After euthanasia, body fluid samples (including blood and urine) were collected and stored at −80 °C. Different tissues/organs (including the brain, heart, liver, spleen, lung, kidney, intestine, fore-limb muscle, hind-limb muscle, spinal cord, thymus, and testis) were aseptically removed, weighed, and homogenized with a mechanical homogenizer (Scientz-192, Scientz, Ningbo, China) in 500 μL PBS and stored at −80 °C prior to virus load assays.

### 2.6. Reverse Transcription and Real-Time PCR

Viral load in the body fluid or solid tissues/organs of infected mice was determined by real-time PCR. Total RNA was extracted from the blood, urine samples, or the individual tissue/organ homogenates using a GenMagSpin Viral DNA/RNA Kit (GenMag Bio, Beijing, China). The RNA was reverse transcribed into cDNA and real-time PCR analysis was performed with a One-Step RT-PCR Kit (GenMag Bio, Beijing, China) according to the manufacturer’s protocol with ZIKV-specific primers (forward, 5′-TGTCTGACAAAGGCTGGAAA-3′ and reverse, 5′-AYGACRAAGTCCCACTCTTGAT-3′) and probe (5′-ROX-ATACAGCTCAGCAGRAAGACTTTTGAGA-BHQ2-3′). Real-time PCR reactions were performed as follows: 42 °C for 15 min and 95 °C for 15 min, followed by 40 cycles of 95 °C for 15 s and 55 °C for 55 s in the LightCycler^®^ 96 real-time PCR system (Roche Applied Science, Penzberg, Germany). A standard curve was generated from 10-fold serially diluted standard samples, a ZIKV fragment plasmid based on the PRVABC59 strain with a correlation coefficient of 1.0, and a PCR efficiency >90% was achieved for each PCR to quantify viral RNA loads in serum, urine, or tissues/organs.

### 2.7. Immunofluorescence (IF) and Immunohistochemistry (IHC) Analysis

Under the condition of anesthetization, mice were humanely euthanized and dissected to isolate and collect organs and tissues (brain, heart, intestine, kidney, liver, lung, limb muscle, spine, testis, and spleen). For histopathological analysis, tissues were fixed with 4% formalin (in PBS) for 72 h at room temperature. Then, the fixed tissues were bisected, embedded in paraffin, and sliced into sections (4 µm thick). The tissue sections were then stained with hematoxylin and eosin (H&E), and the anti-E protein antibody (a monoclonal antibody, 5H1, generated in our laboratory) using the Ultrasensitive TM S-P kit (Fuzhou Maixin Biotechnology Development Co., Ltd., Fuzhou, China) and DAB Detection Kit (Streptavidin-Biotin; Maixin Biotechnology Development Co., Ltd., Fuzhou, China) according to the manufacturer’s protocols.

To examine ZIKV infection in the brain using the immunofluorescence & immunohistochemistry (IF-IHC) method, brain tissues were fixed in 4% paraformaldehyde (PFA) (in PBS) at 4 °C overnight, dehydrated in 30% sucrose (in PBS), and frozen in optimal cutting temperature (OCT) (Leica, London, UK). Sections (15 μm thick) were used for immunofluorescence staining. Sections were incubated with the anti-E protein monoclonal antibody (5H1). Then, goat anti-mouse IgG-Fluorescein (FITC) antibody (#F5387, Sigma, St Louis, MO, USA) was incubated with the tissue sections. The 4′,6-diamidino-2-phenylindole (DAPI) (#D1306, Invitrogen, Carlsbad, CA, USA) was used for visualizing the nucleus. All images of IF-IHC were taken under a Zeiss Axio Imager Z2 microscope (MicroscopeWorld, Carlsbad, CA, USA).

### 2.8. Statistical Analysis

All statistical data were analyzed with GraphPad Prism Software version 5.01 (GraphPad Prism software Inc., La Jolla, CA, USA). Survival curves were compared by the log-rank test. Data are given as mean ± SEM as indicated; “*n*” refers to the sample size.

## 3. Results

### 3.1. One-Day-Old C57BL/6, Kunming (KM) and ICR, Not BALB/c, Were Fatally Susceptible to ZIKV Infection

**Figure 1:**
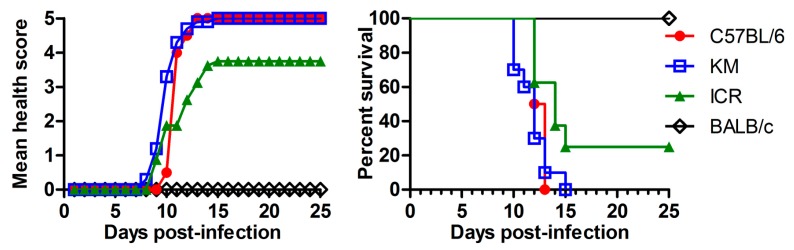
Susceptibilities of different species of neonatal mice to Zika virus (ZIKV) infection. ZIKV (PRVABC59) was used to infect the 1-day-old mouse i.p. (intraperitoneally) at a dose of 10^6^ 50% tissue culture infectious dose (TCID_50_)/mouse in 100 μL volume. Mouse strains were as indicated: BALB/c, C57BL/6, Kunming (KM), and ICR (institute of cancer research). Each group contains 8–10 mice. (**Left**) Health score curve: mice were observed daily for body weight and clinical signs for 25 days after infection or until death. The grade of clinical signs was scored as following: 0, healthy; 1, lethargy and inactivity; 2, wasting; 3, limb weakness; 4, hind-limb or fore-limb paralysis and tremors; and 5, moribund and death; (**Right**) Survival rate curve: the mice were observed for 25 days after infection or until death, and the numbers of deaths and survivors were recorded on a daily base.

Immune-competent adult mice are resistant to ZIKV infection [25,28]. However, as reported previously, one-day-old WT C57BL/6 mice can be infected by ZIKV and have developed disease after infection [44]. We wondered whether other species of neonatal mice can also be infected. In doing so, we infected four species of 1-day-old WT mice—BALB/c, C57BL/6, KM, and ICR—with ZIKV (PRVABC59) i.p. at a dose of 10^6^ TCID_50_/mouse as shown in Figure 1. KM and ICR are the outbred mouse strains, and the BALB/c and C57BL/6 are inbred mice.

As summarized in Figure 1, the ZIKV-infected BALB/c mice did not present any obvious symptoms, and no deaths were observed. KM and C57BL/6 were vulnerable to ZIKV infection; C57BL/6 mice all died within 13 dpi, and KM strain all died within 15 dpi. The susceptibility of ICR mice was lower than KM and C57BL/6, with a lethality of 75%. Since C57BL/6 mice belong to inbred mice and their genotypes are nearly identical, and they are widely used in ZIKV studies, we decided to use C57BL/6 in the following experiments of this study.

**Figure 2:**
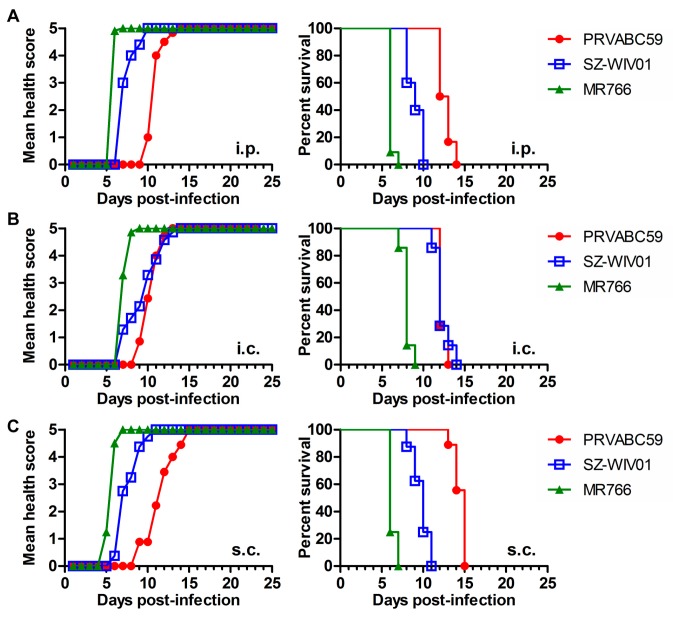
Pathogenic effects of the different strains of ZIKV on 1-day-old C57BL/6 mice via different infectious routes. Different ZIKV strains as indicated were used to infect the 1-day-old mouse at a dose of 10^6^ TCID_50_/mouse i.p. (**A**), intracerebral (i.c.) (**B**), subcutaneously (s.c.) (**C**). Health score curve is shown on the left side while the survival rate curve was shown in right side. Each group contains 8–10 mice.

We used three strains of ZIKV: PRVABC59, SZ-WIV01, and MR766. Both PRVABC59 (isolated in Puerto Rico) and SZ-WIV01 (isolated in China) are Asian strain-derived, and MR766 is the prototype of the African strain. All the strains of viruses were aliquoted, tittered, and stored at −80 °C. The RNA sequencing results showed that the viral RNA genomes were identical to the original ones in https://www.viprbrc.org. To compare the pathogenic effects of different ZIKV strains on neonatal mice, different ZIKV strains (as indicated in Figure 2A–C) were used to infect the 1-day-old C57BL/6 mice via i.p. (Figure 2A), i.c. (Figure 2B), or s.c. (Figure 2C). The infected mice were observed for 25 days or until death. MR766 infection via either i.p., i.c., or s.c. had an 100% mortality rate within 7, 9, and 7 dpi, respectively. PRVABC59 and SZ-WIV01 were uniformly lethal within 15 dpi. In the following experiments of this study, we chose to use PRVABC59 not only because we want to keep in agreement with the previous published study [44] but also because PRVABC59 is an Asian strain that is related to ZIKV-caused microcephaly. We chose i.p. as infection route because it has been used by Lazear et al*.* for the 7-day-old mice [25] and the procedure is less intrusive in neonatal mice.

### 3.2. ZIKV-Caused Lethality of Neonatal Mice Is Age-Dependent of Mouse and Dose-Dependent of Virus

**Figure 3:**
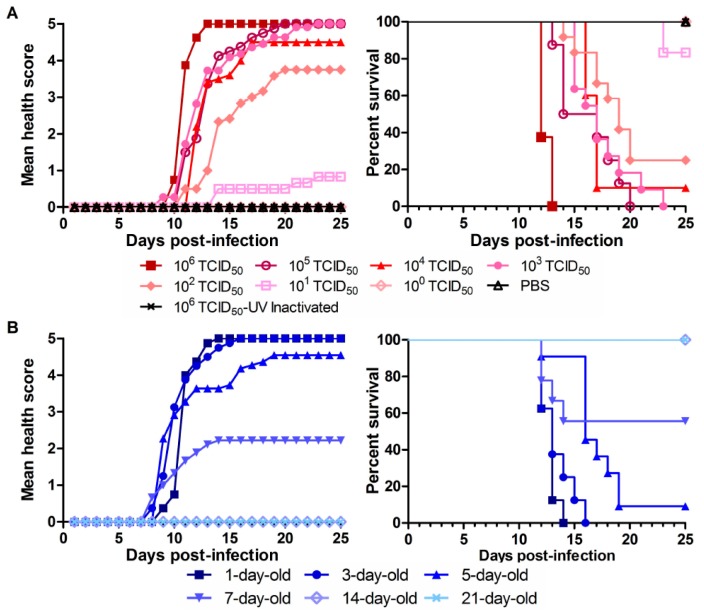
Viral dose-dependent lethality and animal age-dependent lethality by PRVABC59 in neonatal mice. (**A**) A serial 10-fold dilution was made for PRVABC59 stock. One-day-old mice were injected i.p. with PRVABC59 of 10^6^–10^0^ TCID_50_/mouse, or with the UV-inactivated virus of 10^6^ TCID_50_ or PBS; (**B**) Different days old mice (C57BL/6) were infected i.p. with 100 μL PRVABC59 (10^6^ TCID_50_/mouse). All the mice were observed for 25 days after infection or until death. Each group contains 8–10 mice.

Next, we set out to determine the amount of virus that was needed to cause fatal disease in the neonates. To that end, we infected nine groups of 1-day-old mice (8–10 mice per group) i.p. with a different amount of PRVABC59 (10^0^–10^6^ TCID_50_/mouse) or the UV-inactivated virus or PBS as negative control. UV-inactivated viruses were tested on Vero or ARPE19 cells and demonstrated not growing. As shown in Figure 3A, deaths were not seen in groups of PBS, UV-inactivated PRVABC59 or 10^0^ TCID_50_ virus-infected. On the contrary, 100% of mice died within 20 dpi and 13 dpi in the 10^5^ and 10^6^ TCID_50_ virus infected groups, respectively. We calculated the lethality of our PRVABC59 stock in 1-day-old C57BL/6 mice at LD_50_ (i.p.) = 3.72 × 10^1^ TCID_50_.

It has been defined that a neonatal period lasts 28 days in humans and only 7 days in mice [48]. We were curious about whether viral lethality occurs in older newborns. To know that, we infected different ages of neonatal C57BL/6 mice as indicated in Figure 3B. We found that PRVABC59 infection had a 100% mortality rate within 14 and 16 dpi, respectively, in 1- and 3-day-old mice. Five- and 7-day-old mice were still vulnerable to viral infection with a mortality rate of 90.9% and 55.6%, respectively. However, the 14- and 21-day-old mice all survived from viral infection without obvious clinical signs. To know how ZIKV affects development of mice, we chose the 1-day-old mouse in the following experiments.

### 3.3. ZIKV Replicates in Multiple Organs and Tissues of the Infected Neonates

**Figure 4:**
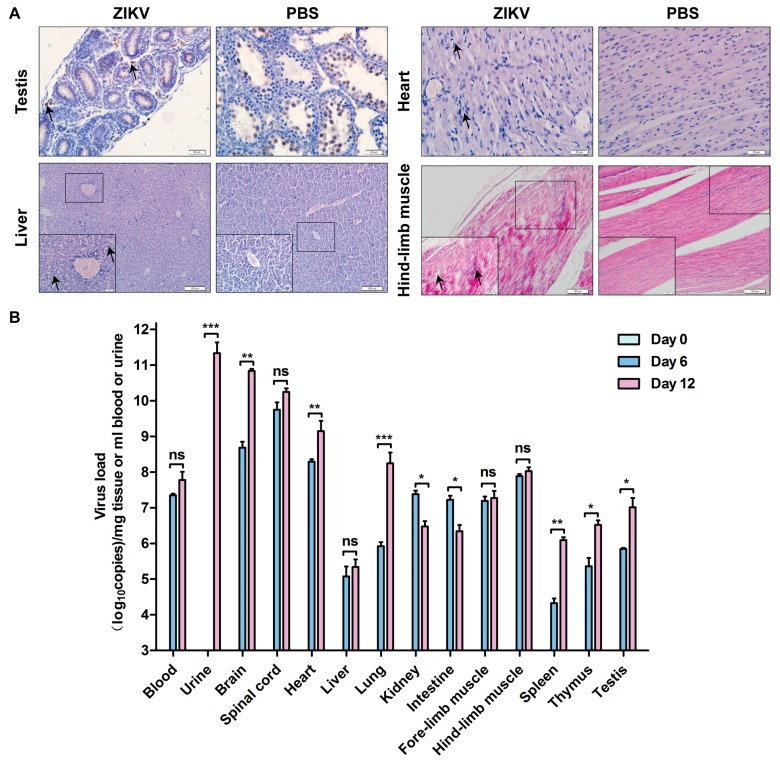
ZIKV infects multiple organs of neonatal C57BL/6 mice. (**A**) Immunohistochemistry (IHC) analysis of tissues after being infected with PRVABC59. The 1-day-old C57BL/6 mice were infected i.p. with 10^6^ TCID_50_ PRVABC59 per mouse or PBS. Mice were sacrificed at 11 dpi to isolate tissues for sectioning and being stained by IHC method. Representative pictures from PRVABC59-infected tissue slides were selected to show: testis, heart, liver, and hind-limb muscle; (**B**) Real time RT-PCR. The 1-day-old mice were infected i.p. with 10^6^ TCID_50_ PRVABC59 per mouse for different days as indicated. The mice were then dissected following euthanasia at 0, 6, and 12 dpi to isolate the organs and tissues as indicated: blood, brain, spleen, heart, intestine, urine, heart, lung, fore-limb muscle, thymus, liver, kidney, hind-limb muscle, and testis. Total RNA samples were prepared using a GenMagSpin Viral DNA/RNA Kit (GenMag Bio, Beijing, China), and real-time RT-PCR was performed to determine the ZIKV RNA level in the isolated or collected organs and tissues. For each length of time kept, we used three mice. The average number of the RNA copies from the real time RT-PCR was obtained statistically with GraphPad Prism Software version 5.01. (the statistical significance was not determined. The graph only showed the average ± S.D.). Unpaired *t* test was used for statistical analysis. * *p* < 0.05, ** *p* < 0.01, *** *p* < 0.001, ns: not significant. Scale bars: A upper 20 μm, A lower 100 μm.

To know whether ZIKV infected different organs and tissues, we infected the 1-day-old mice via i.p. with 10^6^ TCID_50_ PRVABC59 per mouse. The mice were then dissected following euthanasia to isolate the organs and tissues. IHC or H&E were performed to visualize the viral proteins in the tissues as shown in the Figure 4A for testis, heart, liver, and hind-limb muscle. The results were compared with the PBS-treated mice. The ZIKV E protein was detected in brown color in the testis of the PRVABC59-infected mice. H&E staining showed that muscular fiber was damaged, which may be associated with inflammatory response and symptoms. Other pathogenic effects of ZIKV in mice include the vacuolar formation in liver and heart. Indeed, ZIKV replication was obviously seen in all the tissues tested: heart, liver, hind-limb muscle, and testis. Therefore, our experimental results demonstrated that ZIKV infected multiple organs and tissues of the 1-day-old mice.

To know whether ZIKV replicated in different organs and tissues, we infected the 1-day-old mice i.p. with 10^6^ TCID_50_ PRVABC59 per mouse for different days as indicated in Figure 4B. We then dissected the mice following euthanasia at 0, 6, and 12 dpi to isolate and test the organs and tissues including brain, heart, liver, spleen, lung, kidney, intestine, fore-limb muscle, hind-limb muscle, spinal cord, thymus, testis, urine, and blood. The RNA levels in the blood, urine samples or collected organs and tissues were examined by RT-qPCR and were analyzed by comparison with that of Day 0. As shown in Figure 4B, PRVABC59 replicated in all the tested tissues/organs. First, viral replication was detected in most organs and tissues at 6 and 12 dpi, with the highest loads in urine, brain, spinal cord and heart. The late presentation of the viral RNA in the urine, which cannot be detected until 12 dpi, suggested that urination could be a route for excretion and infectious source of virus. Second, ZIKV load in intestine and kidney were reduced at 12 dpi than that of 6 dpi. Importantly, the viral loads increased in the brain, heart lung, spleen, thymus, and testis, implying that ZIKV may cause pathogenesis in these organs. Therefore, our results suggested that ZIKV infects and replicates in multiple organs and tissues to cause diseases in neonatal mice.

### 3.4. ZIKV Infects Multiple Areas of the CNS

**Figure 5:**
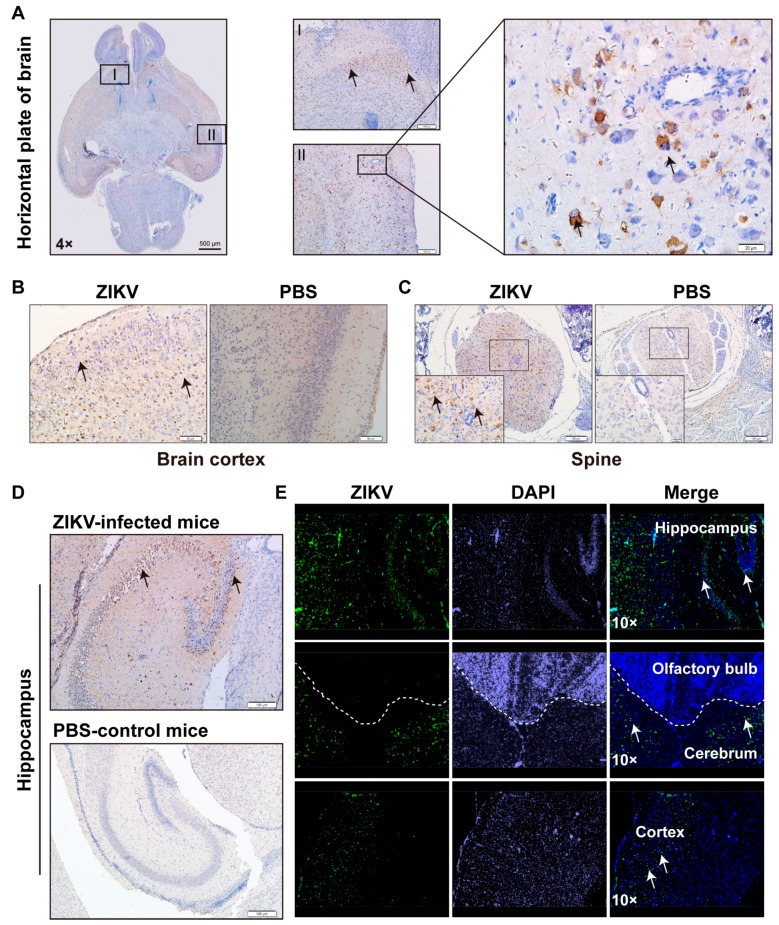
ZIKV infected multiple areas of CNS. (**A**) Horizontal section of the brain of the ZIKV-infected mouse was stained with anti-E protein. The area I and II was amplified to show the infection of ZIKV (brown color stand for the positive infection; (**B**–**D**) Immunohistochemistry (IHC) analysis of CNS tissues after being infected with PRVABC59. The 1-day-old C57BL/6 mice were infected i.p. with 10^6^ TCID_50_ PRVABC59 per mouse or PBS. Mice were sacrificed at 11 dpi to isolate brain tissue for sectioning and staining by the IHC method. Representative pictures from PRVABC59-infected brain tissue slides were selected to show (**B**) brain cortex; (**C**) spine; (**D**) hippocampus; (**E**) the same as in (**B**–**D**) but stained with IFA for hippocampus, olfactory bulb, cerebrum, and cortex. Scale bars: A left 500 μm, A middle, 100 μm, A right, 20 μm; B 50 μm; C 100 μm; D 100 μm.

As we have observed several symptoms, such as unbalanced walking, paralysis, and tremor, we believed that ZIKV replicates and damages the CNS tissues to cause functional abnormalities. To confirm this hypothesis, we performed IHC assay for different areas of CNS tissues for the 1-day-old mice that were injected i.p. with PRVABC59 or PBS. First, we examined different areas of the brain for viral protein expression (E protein) using IHC and/or immunofluorescent assay (IFA) as shown in Figure 5. The 4× microscopic image in Figure 5A showed a horizontal plate of mouse brain at day 11 after infection of ZIKV. Zone I and II, represented cerebrum and cortex region, respectively, were shown at a higher magnification to display clearer IHC results, which showed positive expression of ZIKV E protein. The results demonstrated that these areas were infected with ZIKV (Figure 5A). Then, IHC assays were further performed on the other areas of CNS (such as spine and hippocampus) to examine the ZIKV E protein. Compared to PBS-control mice, obvious signals of ZIKV E protein can be seen as a strong brown staining in Figure 5B for Cortex, Figure 5C for spine, and Figure 5D for hippocampus. We also performed IFA to determine the ZIKV E protein expression in cerebrum, cortex and olfactory bulb, as shown in Figure 5E. Clearly, ZIKV protein was strongly expressed in the cortex and cerebrum, but not in the olfactory bulb. Therefore, we revealed that ZIKV replicates not only in gray matter, but also in hippocampus, cerebrum, and cortex, not in olfactory bulb (Figure 5). Cerebrum and gray matter might be the major sites of ZIKV replication to have a high titer of viral production in brain. Our results suggested that ZIKV infected multiple areas in the brain of neonatal C57BL/6 mice.

### 3.5. ZIKV Replicates in CNS

**Figure 6:**
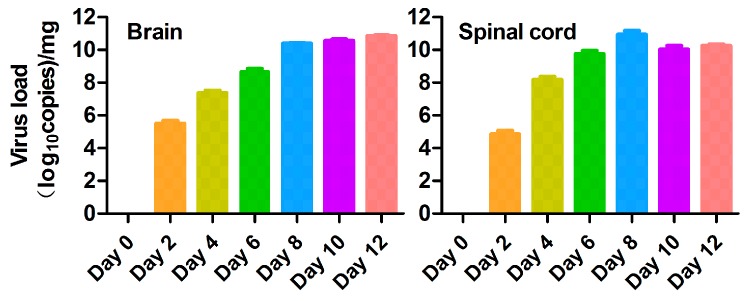
Replicated ZIKV levels in brain and spinal cord of the PRVABC59-infected neonatal C57BL/6 mice. The 1-day-old mice were infected i.p. with 10^6^ TCID_50_ PRVABC59 per mouse for different days as indicated. The mice were then dissected after euthanasia at 0, 2, 4, 6, 8, 10 and 12 dpi to isolate the organs and tissues as indicated: brain and spinal cord. Real time RT-PCR was performed to determine the ZIKV RNA level in the isolated or collected organs and tissues. For each length of time kept, we used three mice. The average number of the RNA copies from the real time RT-PCR was obtained statistically with GraphPad Prism Software version 5.01. (The statistical significance was not determined. The graph only showed the means ± S.D.).

We wondered whether ZIKV replicates in the CNS. We infected the 1-day-old C57BL/6 mice i.p. with 10^6^ TCID_50_ PRVABC59 per mouse for different days as indicated in Figure 6. The mice were then dissected after euthanasia at 0, 2, 4, 6, 8, 10, and 12 dpi to isolate the brain and spinal cord. The RNA levels were examined by RT-qPCR and the viral loads at different time points were shown as Log_10_ copies per mg. As shown in Figure 6, PRVABC59 replicated in the brain and spinal cord tissues. First, viral replication was detected at 2 dpi. The viral load in spinal cord and brain reaches the highest level at 8 and 12 dpi, respectively. This information suggested that ZIKV produces viral particles in brain and spinal cord.

### 3.6. ZIKV Causes CNS Abnormalitiess and Affects Development of Mice

**Figure 7:**
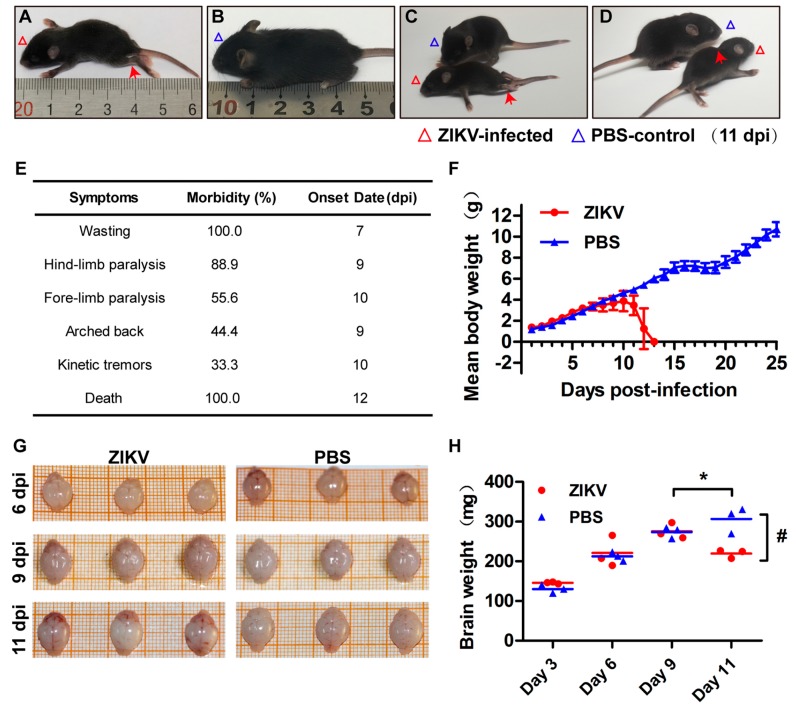
Pathogenic manifestations of PRVABC59 infection in the 1-day-old C57BL/6 mice. The PRVABC59-infected 1-day-old mice were closely observed and typical symptoms were recorded. Representative images at 11 dpi presented as: (**A**) Hind-limb paralysis a; (**B**) PBS-injected mouse control; (**C**) (upper versus lower). PBS-injected control mouse versus wasting and paralysis; (**D**) form an arch on back; (**E**) Symptoms, morbidity rate, and onset date of PRVABC59-infected mice; (**F**) The averaged body weight of ZIKV-infected or PBS-injected Mice (*n* = 8–10 per group); (**G**) Whole brain sizes; (**H**) Brain weights. The averaged brain weight from ZIKV-infected or PBS-injected mice at 3, 6, 9, or 11 dpi (*n* = 3). * *p* < 0.05, the averaged brain weight from ZIKV-infected mice at 9 dpi was compared to that at 11 dpi. # *p* < 0.05, the averaged brain weight from ZIKV-infected mice was compared with that of PBS-injected mice at 11 dpi, all data are means ± S.D., *t*-test.

Every mouse used in our experiments had been carefully observed and compared to the control mouse. We examined the size of the mice as shown in Figure 7A,B and recorded the symptoms of PRVABC59-infected mice (Figure 7E). Compared to control group mice (PBS injected), ZIKV-infected mice showed a smaller weight and body size (Figure 7A,B, or Figure 7F). The symptoms caused by ZIKV in the neonates include hind-limb paralysis (pointed by the red arrow Figure 7A or Figure 7C), wasting and paralysis (Figure 7), forming arched back (pointed by the red arrow in Figure 7D), defect in growth, loss of balance, kinetic tremors, severe ataxia, and death within 15 dpi.

As shown in Figure 7G or Figure 7H, we did not see an obvious difference of the sizes between the PBS-injected and the ZIKV-infected mice at days 6, 9, and 11 post infection. However, the average brain weight from 10 ZIKV-infected mice is significantly lighter than that from the 10 PBS-injected mice at 11 dpi (Figure 7H). In the group of PBS-injected mice, as shown in the right of Figure 7G, the brains weights grew along with the time; however, the brain weights in ZIKV-infected mice stopped growing at 9 dpi because the brain weight in 11 dpi is significantly lighter than that at 9 dpi (Figure 7H). Therefore, ZIKV infection not only affected mice body size, but also brain development.

## 4. Discussion

The recent outbreak of ZIKV has attracted attention worldwide [16,18,34]. The recent reports that ZIKV infection is probably associated with microcephaly of the neonates and GBS in adults spurred researchers to seriously reevaluate the medical significance of this agent as a pathogen of significance to global public health [15,16,17,18]. In general, the majority of ZIKV-infected pregnant women deliver normal babies, but a minority (5%) give birth to abnormal neonates who suffer from permanent congenitally neurological disorders such as microcephaly [49,50]. Although in vitro experiments revealed how ZIKV interacts with cells in cell culture, animal models are needed to identify the factors that are essential for viral pathogenesis. Indeed, recent in vivo studies using mouse models revealed that ZIKV can pass through the placental barrier to infect the fetus and the infected fetus may die or develop microcephaly or other malformations of the brain [23,24,25]. Seven types of adult murine models have been established for ZIKV infection to study pathogenesis, drug development, and vaccine development. These mice include (1) A129 that lacks the *Ifnar1* gene [28,51]; (2) C57BL/6 *Ifnar1−/−* that lacks the *Ifnar1* gene [25]; (3) AG129 that lacks both type I and II IFN receptor genes [23,27,28,52,53,54]; (4) TKO mice with deficient in *Irf3*, *Irf5*, and *Irf7* (*Irf3*^−/−^ *Irf5*^−/−^ *Irf7*^−/−^ triple knockout) [25,55]; (5) SJL mice that are considered as immunocompetent mice [56]; (6) WT C57BL/6 mice treated with a blocking anti-IFNAR1 monoclonal antibody [57]; and (7) the STAT2−/− mice [41]. However, these mouse models mostly use IFN-defective mice that have either IFN receptor deficiency, IFN production deficiency, or IFN-stimulated gene (such as STAT2) deficiency. The SJL mice are IFN-competent, but they were shown to be defective in developing suppressor T cells following stimulation with Con A and have a functional B-cell defect [58]. Most of the mouse models are used to study the congenital infection of ZIKV; it is unknown whether an infection of ZIKV in a newborn baby (a postnatal infection) could cause severe diseases.

Our experimental results showed that ZIKV not only replicated in the 1- to 3-day-old neonatal mice of C57BL/6, or KM species, but also caused death in the mice. The death rate within 15 days post infection was 100% in the 1-day-old C57BL/6 mice. To ensure that our results were accountable, we selected four different species of mice: two inbred mice (C57BL/6 and BALB/c) and two outbred mice (KM and ICR). We found that ZIKV can cause 100% lethality in 1-day-old C57BL/6 and KM, 75% in ICR, but no deaths were seen in BALB/c (Figure 1). Despite death not being observed in the BALB/c mice, other events may have been overlooked. We did not perform a viral titer examination for the ZIKV-infected BALB/c mouse, so we do not exclude the possibility that ZIKV could replicate in BALB/c without lethality. Interestingly, a recent study showed that three ZIKV strains (MTQ/2015, VEN/2016, and SAM/2016), but not their ancestor (CAM/2010) caused mortality in BALB/c mice [59]. We will investigate our hypothesis that neonatal BALB/c mice might have a unique defense arm against ZIKV infection.

In addition, we infected 1-day-old C57BL/6 mice with three different strains of ZIKV (MR766, PRVABC and SZ-WIV01) via i.p., s.c., or i.c. injection and found that all three ZIKV strains can cause 100% lethality within 15 dpi (Figure 2). To keep the parameters of our experiments in agreement with those in the previous experiments, we selected the mouse of 1-day-old neonatal C57BL/6 and the virus of PRVABC59. The viral dose and animal age are also important for ZIKV pathogenesis in neonatal mice as we showed that the ZIKV-caused lethality is viral dose- and animal age-dependent (Figure 3). The mice who survived from ZIKV infection did not present obvious clinical signs in this study. However, the RNA load of the virus and the neutralizing antibody level in the mice need to be determined in the future.

Congenital ZIKV infection interferes with neuronal stem cell (NSC) proliferation and is associated with neuronal disorders [19,60]. ZIKV infection in human adults usually results in mild symptoms, if any at all, such as so-called Zika fever. These symptoms have been also seen in adult mouse models. Can ZIKV impose a threat to neonates if the infection occurs after birth? A neonatal mouse model might be useful to uncover whether and how ZIKV can infect newborns to cause disease. ZIKV infection in neonatal mice has been reported previously by different groups [25,27,44,61].

Our present studies systemically investigated ZIKV infection in neonatal mice and obtained new insight into ZIKV pathogenesis. First, we observed that ZIKV replicated in CNS, resulted in a high level of viral production in brain and spinal cord, and caused CNS abnormalities in neonatal C57BL/6 mice. These are consistent to the observation of Fernandes et al. in a neonatal Swiss mouse model [61]. In addition, we found that the ZIKV-caused lethality was viral dose-dependent (Figure 3), which implies that neonatal mouse might have developed defense against lower amount infected ZIKV. Lastly, we showed that ZIKV infection had a lethality of 100% in 1- to 3-day-old neonates, but only 55.6% in 7-day-old ones (Figure 3). This was consistent with the report of Lazear et al. [25]. From these experimental results and given that IFNs are the important host defense against ZIKV, we hypothesize that 7-day-old mouse may generate significantly higher levels of IFN. We will test our hypothesis in the future. The other factors such as different strains of ZIKV and inoculation routes might also affect the results of ZIKV infection in the neonatal mice. All the studies showed that ZIKV-infected neonatal mice developed neuronal disorders. Our results showed that ZIKV infection significantly affected the sizes and weights of the brains and the virus replicated strongly in the brain tissues (Figure 6 and Figure 7). The reduction of brain weight by ZIKV infection could be caused by virus-induced cell death. The viral titer reached high levels in multiple organs/tissues (Figure 4) and the virus could be seen in different areas of CNS except the olfactory bulb. Although our experiments showed that the olfactory bulb was not infected by PRVABC59 ZIKV, the Qin group observed that ZIKV strain VEN/2016 significantly infected olfactory bulb of neonatal mice [43,59]. Therefore, the reason why the olfactory is less susceptible to ZIKV infection is more complicated than we thought. Interestingly, we observed that ZIKV infected and replicated in multiple organs and tissues, which might finally have resulted in the deaths of mice. This suggests that the deaths of the neonatal mice by ZIKV might be caused by functional failure of multiple organs.

In summary, we developed a neonatal murine model for ZIKV studies. The neonatal mice showed 100% lethality by different ZIKV strains. A neonatal infection by ZIKV differs from the congenital ZIKV infection in terms of brain development, in that ZIKV infection of neonatal mice did not cause microcephaly. Rather, the ZIKV infection in the neonatal mice caused multiple organ failures. Our data raise an important implication that postnatal ZIKV infection might cause severe disease in the neonates. We plan our future research using the neonatal mouse models to (1) discover why the ZIKV did not infect the olfactory bulb; (2) determine whether BALB/c has a different defense arm against ZIKV from C57BL/6; and (3) ascertain whether ZIKV specifically causes cardiovascular pathogenesis.
